# A systematic review of randomized clinical trials on quadrivalent influenza vaccines for adults: Andrea Pellacchia

**DOI:** 10.1093/eurpub/ckac129.599

**Published:** 2022-10-25

**Authors:** A Pellacchia, A Mannocci, R Millevolte, M Chiavarini, C de Waure

**Affiliations:** 1 Department of Medicine and Surgery, University of Perugia, Perugia, Italy; 2 Faculty of Economics, “Universitas Mercatorum” University of Rome, Rome, Italy

## Abstract

**Background:**

Vaccination is the most effective intervention to prevent influenza. Adults at risk of complications are among the targets of annual vaccination campaigns and can receive different types of quadrivalent influenza vaccines (QIV). To assess the immunogenicity of different QIVs we performed a systematic review of available randomized controlled trials (RCTs) with the aim of indirectly compare them through a network meta-analysis.

**Methods:**

The systematic review was conducted in accordance with PRISMA-NMA guidelines. We systematically searched RCTs conducted in adults aged 18-64 years that assessed the immunogenicity, namely seroprotection and seroconversion rate (SPR and SCR), of any QIV compared to any comparator. The literature search was performed on three databases (Medline, Cochrane Library and Scopus) until March 30th, 2021.

**Results:**

Twenty-four RCTs were included in the systematic review. A network meta-analysis was not possible: the assumption of transitivity was not satisfied. Therefore, we decided to combine data on immunogenicity and efficacy of each QIV through single meta-analyses in the presence of at least two studies. Live attenuated QIV showed the worst results in terms of both SCR and SPR. Standard dose egg based, low dose adjuvanted, cell based, recombinant and intradermal QIV showed similar SCR in respect to influenza strain A, whereas low dose adjuvanted QIV showed an overall better profile in respect to B lineages. Regarding SCR, the better results were issued by standard egg based, cell based, recombinant, and low dose adjuvanted QIVs.

**Conclusions:**

Albeit an indirect comparison among different QIVs was not possible, the assessment of SCR and SPR provided an overview of their respective potentiality and criticality in adulthood. In particular attention should be paid to new generation influenza vaccines that can have an antigen sparing effect and to the collection of real world data to make comparison among different QIVs possible.

**Key messages:**

• Available evidence does not allow to perform indirect comparison of the quadrivalent influenza vaccines for adults.

• Some QIVs, including new generation ones, elicit a better antibody response.

